# The neurodevelopmental precursors of altruistic behavior in infancy

**DOI:** 10.1371/journal.pbio.2005281

**Published:** 2018-09-25

**Authors:** Tobias Grossmann, Manuela Missana, Kathleen M. Krol

**Affiliations:** 1 Department of Psychology, University of Virginia, Charlottesville, Virginia, United States of America; 2 Max Planck Institute for Human Cognitive and Brain Sciences, Leipzig, Germany; 3 Institute of Educational Sciences, University of Leipzig, Leipzig, Germany; INSERM–CEA Cognitive Neuroimaging Unit, France

## Abstract

Altruistic behavior is considered a key feature of the human cooperative makeup, with deep ontogenetic roots. The tendency to engage in altruistic behavior varies between individuals and has been linked to differences in responding to fearful faces. The current study tests the hypothesis that this link exists from early in human ontogeny. Using eye tracking, we examined whether attentional responses to fear in others at 7 months of age predict altruistic behavior at 14 months of age. Our analysis revealed that altruistic behavior in toddlerhood was predicted by infants’ attention to fearful faces but not happy or angry faces. Specifically, infants who showed heightened initial attention to (i.e., prolonged first look) followed by greater disengagement (i.e., reduced attentional bias over 15 seconds) from fearful faces at 7 months displayed greater prosocial behavior at 14 months of age. Our data further show that infants’ attentional bias to fearful faces and their altruistic behavior was predicted by brain responses in the dorsolateral prefrontal cortex (dlPFC), measured through functional near-infrared spectroscopy (fNIRS). This suggests that, from early in ontogeny, variability in altruistic helping behavior is linked to our responsiveness to seeing others in distress and brain processes implicated in attentional control. These findings critically advance our understanding of the emergence of altruism in humans by identifying responsiveness to fear in others as an early precursor contributing to variability in prosocial behavior.

## Author summary

Altruistic behavior such as helping an unfamiliar person in need is considered a key feature of cooperation in human societies. Yet our propensity to engage in altruistic acts varies considerably among individuals, ranging from extraordinarily altruistic kidney donors to highly antisocial psychopaths. We examined the hypothesis that our responsiveness to seeing others in distress (displaying fear) accounts for variability in helping behavior from early in development. Confirming this hypothesis, our results demonstrate that attentional and brain responses measured when viewing fearful faces, but not happy or angry faces, at 7 months predicted helping behavior at 14 months of age. Specifically, heightened initial attention to fearful faces followed by enhanced disengagement from fearful faces in infancy was associated with greater helping behavior in toddlerhood. These findings advance our understanding of human altruism by identifying responsiveness to fearful faces as an early precursor to concern for others reflected in helping behavior. This lends support to the view that social competencies that facilitate cooperation emerge early in human ontogeny.

## Introduction

Why humans display altruistic behaviors towards genetically unrelated individuals is one of the most enduring questions in biology and psychology [1]. From a phylogenetic perspective, altruistic behaviors are not unique to humans but are also found in other animals, including our closest living relatives, the chimpanzees [2, 3]. From an ontogenetic perspective, altruistic behavior emerges early in human development. For instance, infants have been shown to help others in need already at the young age of 14 months [2]. Based on these findings, it has been argued that it is in our nature to be altruists [4, 5].

Yet the tendency of humans to engage in altruism varies considerably across individuals [6]. In fact, extreme cases with regard to this tendency exist, ranging from extremely prosocial anonymous kidney donors to highly antisocial psychopaths [7, 8]. The study of these extreme cases informs the question of what contributes to individual differences in altruism and also provides insights into the foundations of altruistic behavior more generally. Specifically, an individual’s responsiveness when viewing others in distress (displaying fear) appears to be a key process related to altruistic tendencies, with kidney donors showing heightened and psychopaths showing decreased sensitivity to fearful faces [7, 8]. At the level of the brain, the amygdala shows diminished responses to fearful faces in psychopaths and enhanced responses in highly altruistic kidney donors [6]. Crucially, these effects are specific to fearful faces, as no such differences are evident in response to other negative emotions such as anger, which signals interpersonal threat (aggression) rather than distress to another person [6]. Enhanced recognition of fear from faces is also associated with higher levels of prosocial behavior among neurotypical adults [9, 10]. Moreover, heightened sensitivity to fearful faces has been linked to greater levels of altruistic behavior in 5-year-old children in 2 different cultures [11]. In this study, children who were quicker to orient to fearful faces displayed greater prosocial behavior in a dictator game (sharing more valuable resources with an unfamiliar peer). Together, these studies suggest that greater sensitivity to fearful faces is linked to heightened levels of prosocial behavior, which can already be seen in preschool children.

The ability to detect and sensitively respond to fear emerges during the first year in infancy [12], which is before the age at which helping behavior is first observed in experimental contexts in toddlers—14 months [2]. In particular, by around 7 months of age, but not younger, human infants show increased neural and behavioral (attentional) responses to fearful faces and discriminate them from other positive and negative facial expressions [13–17]. The widely open eyes, exposing larger amounts of white sclera than for other facial expressions, are the distinguishing facial feature that primarily accounts for infants’ sensitivity to fearful faces [14, 15, 17]. Given the research evidence summarized above, infancy is considered a sensitive developmental period during which fearful face processing skills come online. Examining responsiveness to fearful faces and its variability during this important period in ontogeny represents a unique opportunity to shed light on the precursors of altruistic behavior in early human development. This is because responsiveness to fear in others may be an early emerging marker of (or precursor to) concern for others linked to prosocial tendencies in humans [18].

In the current longitudinal study, we therefore assessed infants’ attentional and neural responses to fearful, angry, and happy faces using eye tracking and functional near-infrared spectroscopy (fNIRS) at 7 months of age and their altruistic behavior in 2 helping tasks at 14 months of age. This allowed us to assess the following main hypothesis. If responsiveness to fearful faces indeed plays an important role in accounting for differences in altruistic behavior in humans, then responding to fearful faces in infancy should longitudinally predict helping behavior in toddlerhood. More specifically, based on prior work with adults and preschool children [8, 11], we hypothesized that responsiveness to fearful faces but not to angry and happy faces at 7 months predicts helping behavior at 14 months. As in prior work with preschool children, we used eye tracking to assess variability in infants’ attention to facial expressions. More specifically, we presented 7-month-olds with images of emotional facial expressions (happy, angry, and fearful faces) always paired with a neutral facial expression in order to determine how much attention is allocated to either of the expressions when compared to a neutral face. Using fNIRS, which preceded the eye tracking, enabled us to examine the brain processes that correlate with infants’ sensitivity to fearful faces and its potential link to prosocial behavior. Our fNIRS experiment was based on a previously published fNIRS study on emotional face processing with infants using happy and angry faces [19], but we critically modified and extended this prior work by including fearful faces and measuring responses from additional brain regions, including regions in the frontal cortex. Our analysis specifically focused on responses within brain regions in the superior temporal, inferior frontal, and dorsolateral frontal cortices (see Materials and methods). These brain regions have respectively been implicated in detecting emotional and intentional states from dynamic facial information [20], in affect sharing [21], and in cognitive control/emotion regulation [22]. In this context, it is also important to mention that fNIRS is limited to measuring cortical responses [23], preventing us from directly measuring amygdala responses found to be sensitive to fearful face processing in adults. However, the cortical brain regions examined in the current study have also been shown to be critically involved in emotional face processing and are interconnected with the amygdala [24, 25].

Moreover, it is important to discuss the existing yet scarce developmental work linking brain processes to prosocial behavior. Prior work in this area has exclusively relied on electro-encephalography (EEG)-based brain measures during rest [26] or in response to watching helping or hindering actions through animated character interactions [27] with older age groups than in the current study. For example, EEG responses linked to controlled rather than early automatic brain processes elicited when discriminating between helping and hindering actions predicted levels of prosocial behavior displayed in the Dictator Game among 3- to 5-year-old children [27]. In a follow-up study based on these findings, it was shown that controlled brain processes evoked over the dorsolateral prefrontal cortex (dlPFC) were associated with a visual preference for helping characters in 1- to 2-year-olds [28]. Interestingly, this study also showed that parental values were associated with children’s preference for prosocial (helping) characters, suggesting an influence of socialization on prosocial preferences. In fact, there is emerging evidence that socialization through reciprocal social interaction and explicit scaffolding (praise and encouragement) may play a role in contributing to toddlers’ displays of prosocial behavior [29, 30].

**Fig 1:**
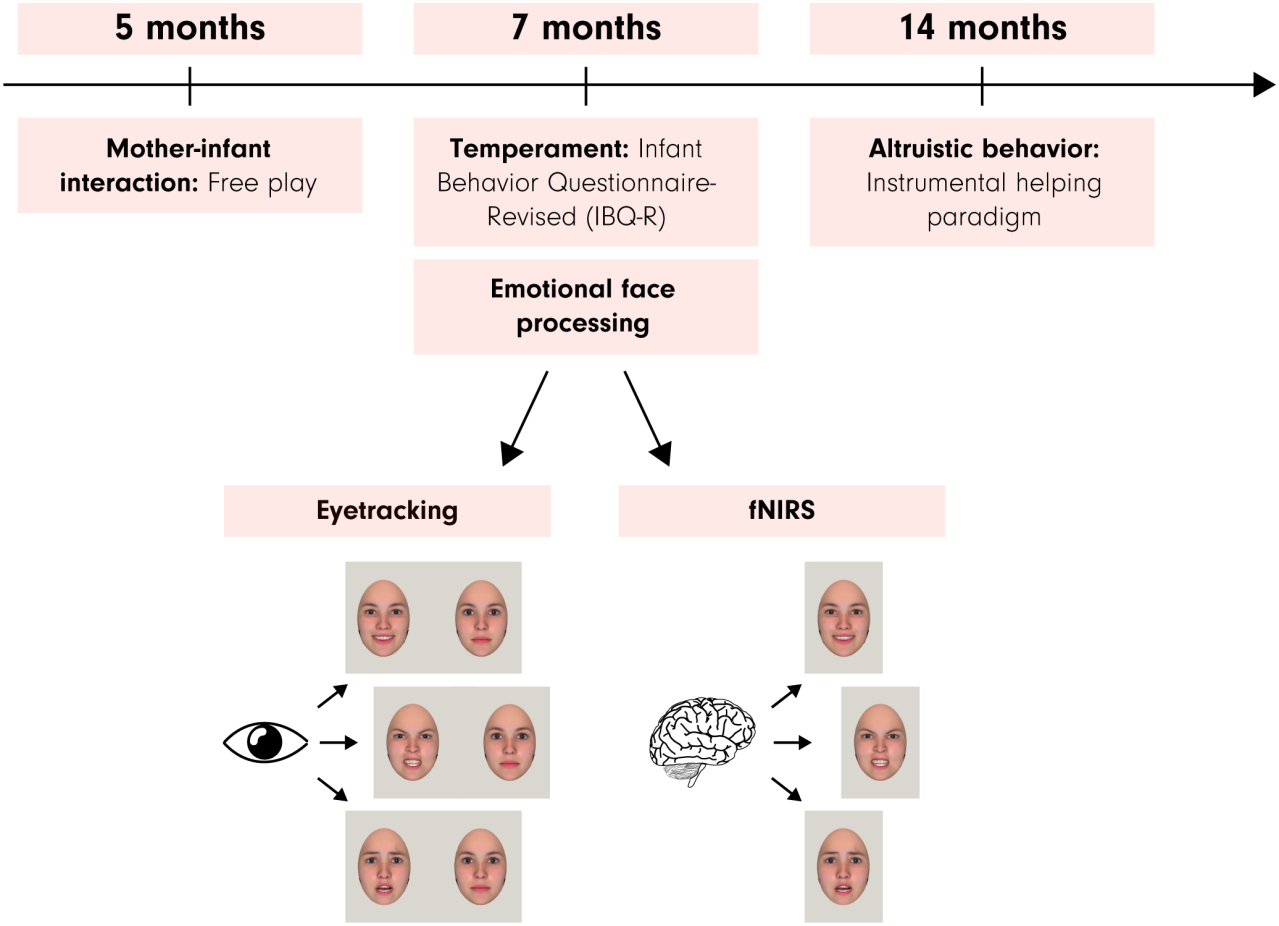
This figure shows an overview of the study design and the methods used with respect to the age of the infant. Please note that infants viewed photographic images of real faces (see Materials and methods) and that the facial images shown here were computer generated with the FaceGen software (https://facegen.com) for illustrative purposes. fNIRS, functional near-infrared spectroscopy.

The current study critically extends previous work and sets itself apart by (a) testing a specific prediction regarding the role of responsiveness to fearful faces in contributing to altruistic tendencies, (b) focusing on the earliest ages when sensitivity to fearful faces and altruistic helping behavior first emerge in development, and (c) measuring attentional responses to emotional faces using eye tracking and examining its neural correlates by recording brain responses using fNIRS (see Fig 1 for an overview of the study design). In addition, given the mentioned emerging evidence concerning the role of socialization and experience in the early development of helping behavior, we decided to explore the possibility that patterns of maternal behavior (measured by coding of social engagement with the infant during free play) may influence altruistic behavior and its brain correlates.

## Results

### Eye tracking

**Fig 2:**
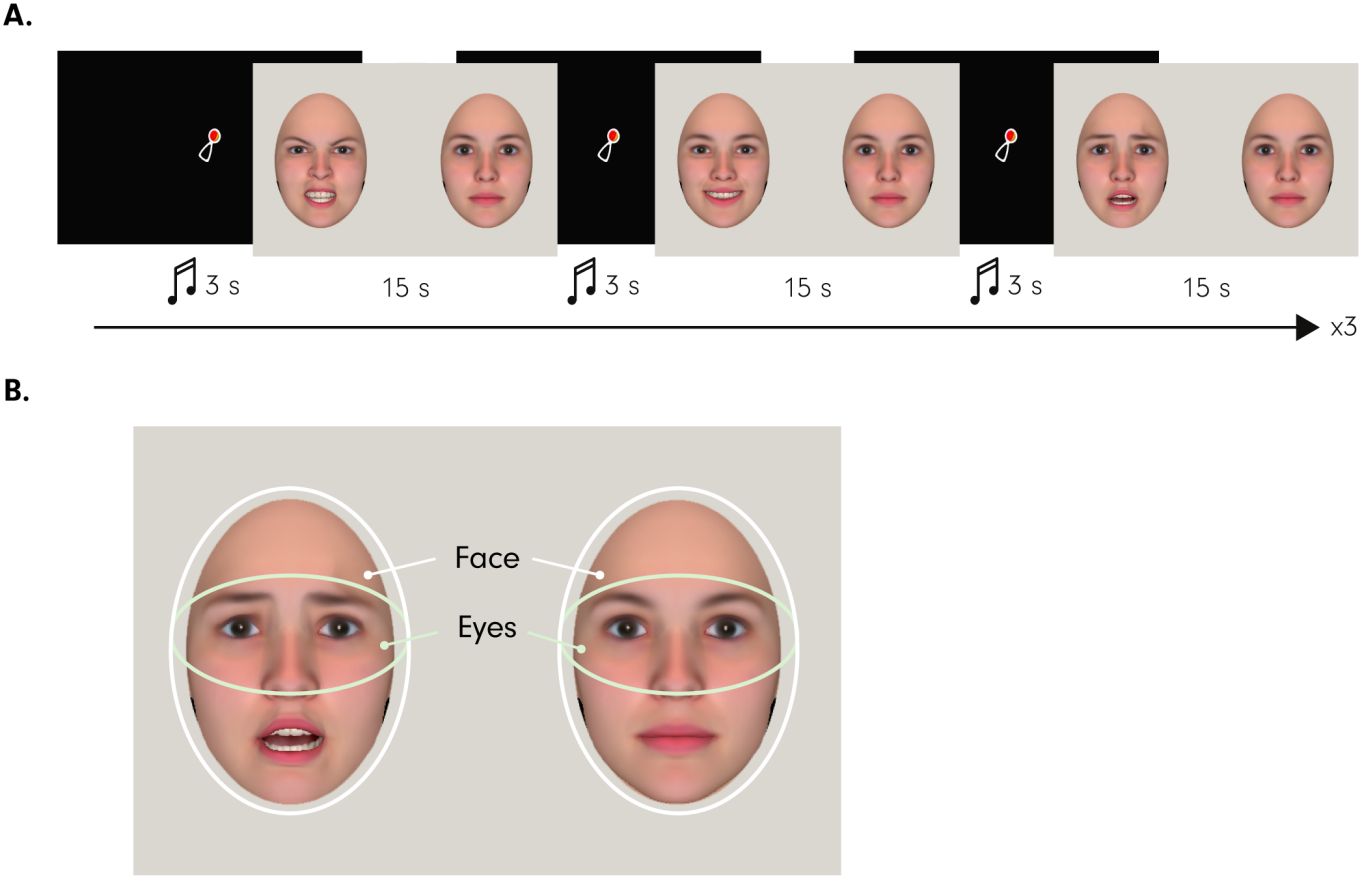
A: This illustrates the eye tracking paradigm employed in the current study at age 7 months. Note that the eye tracking experiment followed the fNIRS experiment. As shown here, infants were presented with experimental trials consisting of a side-by-side presentation of a neutral face and an emotional (happy, angry, or fearful) face. Note that in the actual experiment, the side on which an emotional face and neutral face were presented was counterbalanced, and the face identity changes were pseudorandomized (see Materials and methods for details). B: This illustrates the 2 areas of interest (face and eyes) that were used in the analysis of the eye tracking data. Please note that infants viewed photographic images of real faces (see Materials and methods) and that the facial images shown here were computer generated with the FaceGen software (https://facegen.com) for illustrative purposes. fNIRS, functional near-infrared spectroscopy.

**Fig 3:**
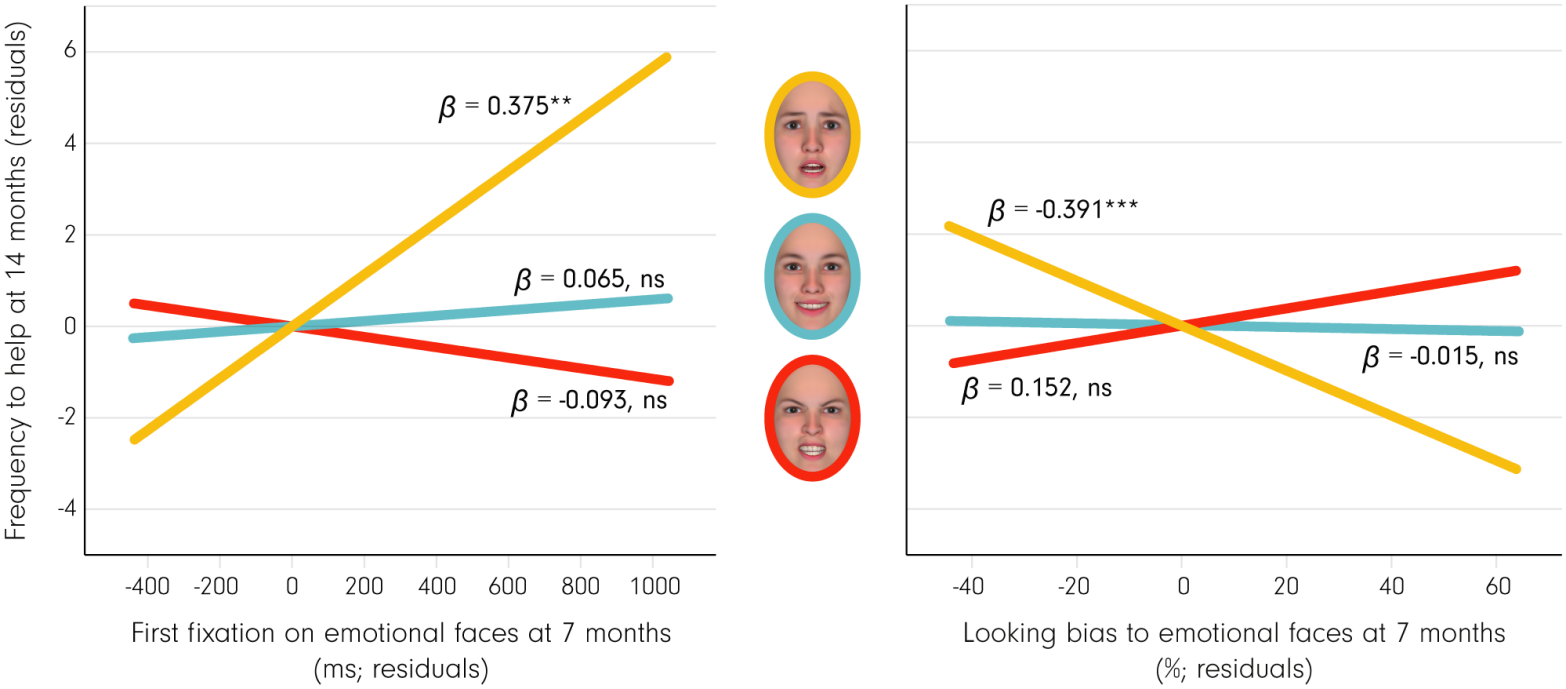
Attention to fearful faces, but not happy or angry faces, predicts altruistic behavior. This shows partial regression plots from a multiple regression illustrating the specific impact of duration of first fixation on a fearful face when compared to the other facial expressions presented in the experiment (left) and looking bias to fearful faces when compared to the other facial expressions presented in the experiment (right) in predicting altruistic behavior measured as frequency of helping at 14 months (plotted on the y-axis; residuals). Please note that infants viewed photographic images of real faces (see Materials and methods) and that the facial images shown here were computer generated with the FaceGen software (https://facegen.com) for illustrative purposes. Underlying data are available through the Open Science Framework (https://osf.io/znjr7/). ns, not significant.

We conducted stepwise multiple regression analyses to test the hypothesis that attention to fearful faces (as measured by duration of first look and overall looking bias), but not happy and angry faces, at 7 months predicts altruistic helping behavior at 14 months (see Fig 2 for the eye tracking methods used, S1 Fig for information regarding the distribution of eye tracking measures, and S1 Table for a group comparison between emotions). Helping behavior was measured as a continuous variable ranging from 0 to 6, with 0 reflecting that the infant did not display any helping behavior and with 6 reflecting that the infant helped in all 6 helping trials. Our analysis including all 3 emotions in the regression model confirmed our hypothesis by showing that the duration of the first look to fearful faces predicted altruistic helping behavior, with increased durations predicting higher levels of altruistic helping behavior, *ß* = 0.362, *t* = 3.005, *p* = 0.004; *R*^*2*^ = 0.131 (first look to angry and happy faces: *ß* = −0.092, *t* = −0.750, *p* = 0.456, and *ß* = 0.063, *t* = 0.523, *p* = 0.603, respectively) (see Fig 3 and Table 1). Furthermore, reduced overall attention to fearful faces, but not happy or angry faces, as measured by the bias to look at fearful faces as compared to neutral faces (see Materials and methods), significantly predicted greater altruistic helping behavior at 14 months, *ß* = −0.404, *t* = −3.481, *p* = 0.001; *R*^*2*^ = 0.163 (attention to angry and happy faces: *ß* = 0.154, *t* = 1.329, *p* = 0.189, and *ß* = −0.032, *t* = −0.269, *p* = 0.788, respectively) (see Fig 3). Our analyses also show that initial attentional engagement with fearful faces (the duration of the first look) and overall attention (looking bias), when entered together into a multiple regression model, explain more than 28% of the variance in altruistic behavior at 14 months, *F*(2, 59) = 11.514, *p* = 0.00006; *R*^*2*^ = 0.281.

**Table 1:** This is an overview of the eye tracking and fNIRS findings when altruistic behavior is measured as a continuous variable (frequency of helping, ranging from 0 to 6). Note that a positive *ß* value reflects that this variable is positively associated with altruistic behavior. Underlying data are available through the Open Science Framework, https://osf.io/znjr7/.

Predictor variables at 7 months	Altruistic behavior as a continuous measure at 14 months
**Eye tracking**	
first look fearful faces	*ß* = 0.362, *p* = 0.004
looking bias fearful faces	*ß* = −0.404, *p* = 0.001
looking bias fearful eyes	*ß* = −0.319, *p* = 0.01
**fNIRS**	
left dlPFC fearful faces	*ß* = −0.272, *p* = 0.037*
right dlPFC fearful minus angry faces	*ß* = −0.335, *p* = 0.008

*Note that this reflects the value when controlling for maternal engagement and that left dlPFC to fearful faces also predicted looking bias to fearful faces.

Abbreviations: dlPFC, dorsolateral prefrontal cortex; fNIRS, functional near-infrared spectroscopy.

When focusing our analysis on the eye region rather than the face as a whole (see Materials and methods), using stepwise multiple regressions, our data revealed that, similar to when using the whole face, overall attention (looking bias) to fearful eyes but not to angry or happy eyes significantly predicts helping behavior, *ß* = −0.319, *t* = −2.65, *p* = 0.01; *R*^*2*^ = 0.102 (attention to angry and happy eyes: *ß* = 0.061, *t* = 0.50, *p* = 0.619, and *ß* = −0.006, *t* = −0.05, *p* = 0.96, respectively) (see Table 1). While the duration of the first look to fearful eyes showed a similar pattern (longer looks, greater helping frequency) as when assessing the first look to the whole face, it failed to significantly predict helping behavior, *ß* = 0.187, *t* = 1.464, *p* = 0.148.

**Fig 4:**
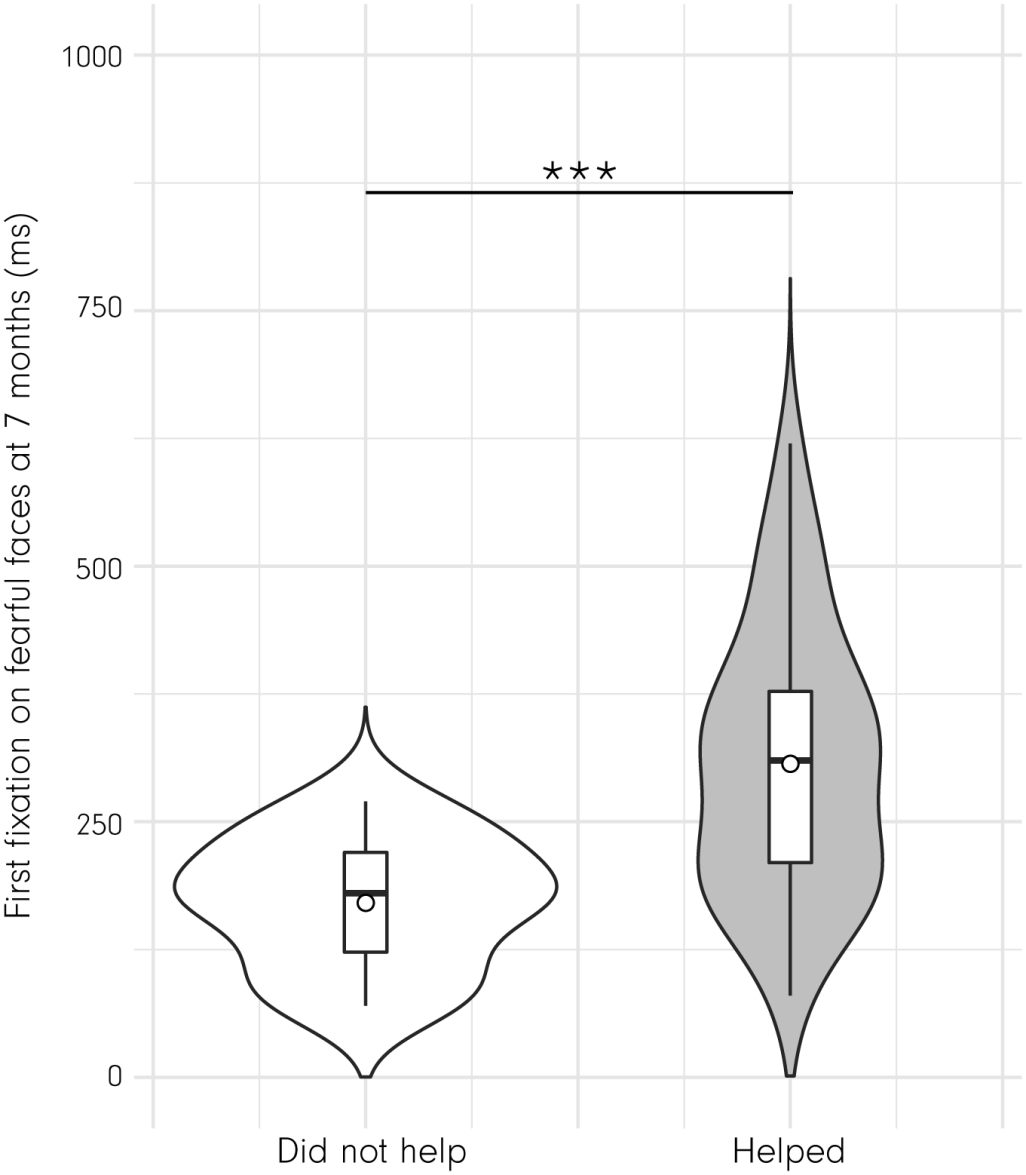
This combined violin–box plot shows that the duration of the first fixation on fearful faces at 7 months of age was significantly longer in infants who helped at age 14 months compared to infants who did not help (using altruistic behavior as a binary measure). Note that the duration of the first fixation on fearful faces at 7 months significantly predicts altruistic behavior at 14 months in a logistical regression (see Results and Table 2). *** *p* < 0.001. Underlying data are available through the Open Science Framework, https://osf.io/znjr7/.

As an additional analysis, we explored whether the obtained results hold when helping behavior is coded as a binary measure (helping versus no helping) rather than as a continuous measure, as was done in the analysis presented above (see Table 2). A logistical regression analysis, with the binary helping measure (helping versus no helping) as an outcome, revealed that the duration of the first look to fearful faces significantly predicted helping behavior, *b* = 14.579, χ^*2*^ *(1) =* 21.255, *p* = 0.000004; *R*^*2*^ = 0.399 (see Fig 4). This pattern was confirmed when using the duration of the first look to fearful eyes instead of faces as a predictor for helping behavior in a logistic regression model, *b* = 6.645, χ^*2*^ *(1) =* 8.199, *p* = 0.004; *R*^*2*^ = 0.166. When overall attention (looking bias) to fearful faces was used as a predictor in a logistical regression predicting the binary helping outcome, it just failed to reach significance, *b* = −3.043, χ^*2*^ *(1) =* 3.065, *p* = 0.08 (predicting by looking bias to fearful eyes was not significant, *p* = 0.213). These results from the logistical regression model using a binary helping measure principally confirm the results from our analysis using a continuous measure of helping and point to an initial heightened attention (first look) to fearful faces and eyes as the best predictor of whether an infant does or does not help at age 14 months.

**Table 2:** This is an overview of the eye tracking and fNIRS findings when altruistic behavior is measured as a binary variable (help versus no help). Note that a positive *b* value reflects that this variable is positively associated with altruistic behavior. Underlying data are available through the Open Science Framework, https://osf.io/znjr7/.

Predictor variables at 7 months	Altruistic behavior as a binary measure at 14 months
**Eye tracking**	
first look fearful faces	*b* = 14.579, *p* = 0.000004
first look fearful eyes	*b* = 6.645, *p* = 0.004
**fNIRS**	
right dlPFC fearful faces	*b* = −0.142, *p* = 0.022

Abbreviations: dlPFC, dorsolateral prefrontal cortex; fNIRS, functional near-infrared spectroscopy.

Note that all reported effects of the eye tracking measures on prosocial behavior remain significant when adjusting the *p-*value threshold to *p* < 0.01.

In summary, our eye tracking results clearly show a specific effect of attention to fearful faces on altruistic tendencies in early development. Our results index a pattern of initial increased attention to fearful faces and eyes, followed by reduced attentional bias to (enhanced disengagement from) fearful faces and eyes being associated with greater levels of altruistic behavior.

### fNIRS

**Fig 5:**
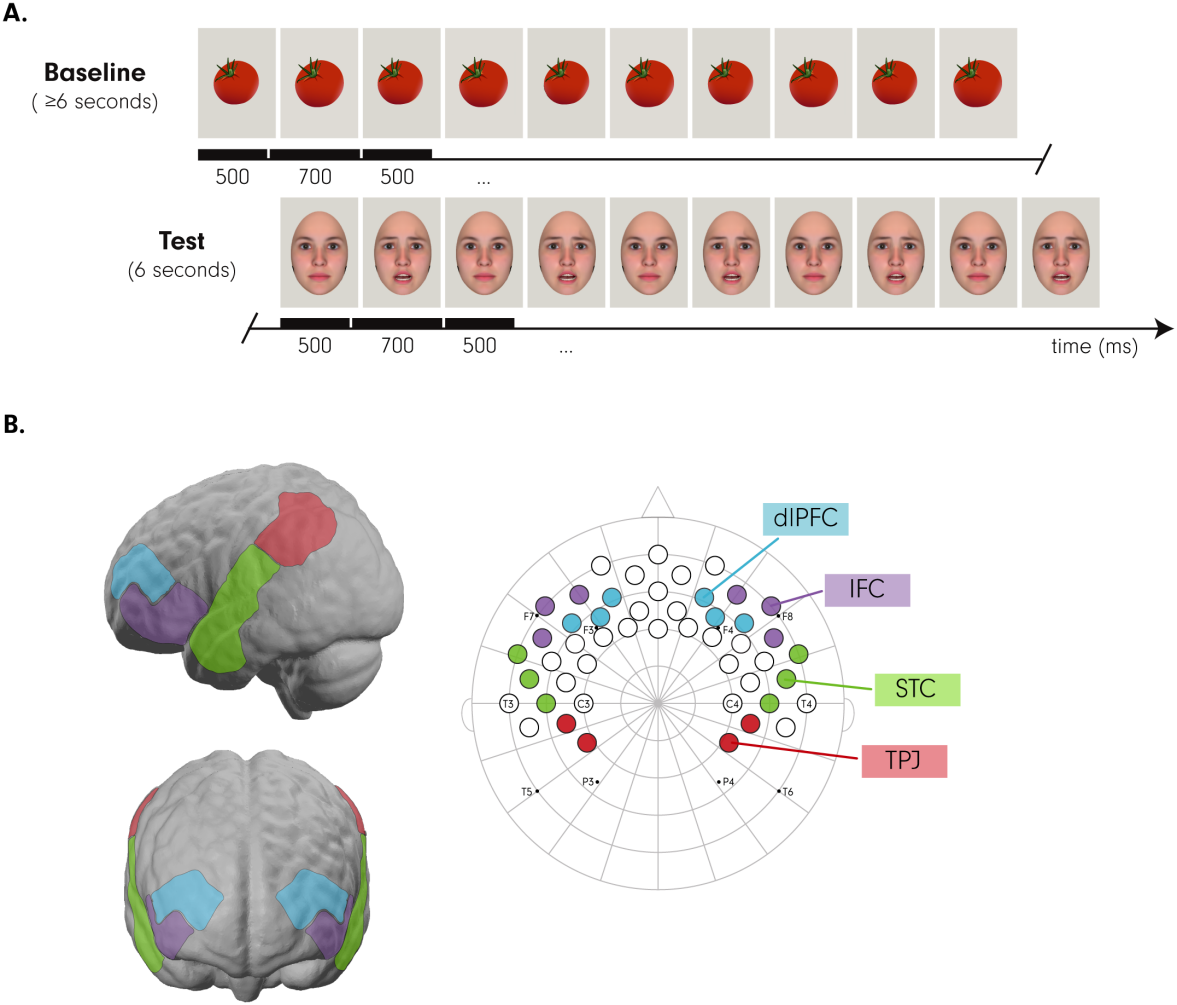
A: This illustrates the fNIRS paradigm employed in the current study at age 7 months. Note that the fNIRS experiment preceded the eye tracking experiment. Infants viewed at least 6 seconds of nonsocial (inanimate) baseline stimuli followed by 6 seconds of emotional (happy, angry, and fearful) test stimuli. Dynamically changing stimuli were administered by presenting neutral faces that rapidly and repeatedly changed to one of the 3 emotions. B: This shows the fNIRS channel layout (right) with the nose plotted up and the 3 ROIs marked by different colors (dlPFC = blue; IFC = violet; STC = green; TPJ = red) on the left with reference to the 10–20 system commonly used in EEG research. On the left, these ROIs are also shown when projected onto the cortical surface of a frontal-view (bottom) and lateral-view brain (top). Please note that infants viewed photographic images of real faces (see Materials and methods) and that the facial images shown here were computer generated with the FaceGen software (https://facegen.com) for illustrative purposes. EEG, electro-encephalography; fNIRS, functional near-infrared spectroscopy; IFC, inferior frontal cortex; ROI, region of interest; STC, superior temporal cortex; TPJ, temporo-parietal junction.

We conducted a stepwise regression analysis to explore which a priori defined brain regions of interest (superior temporal cortex, inferior frontal cortex, and dlPFC in both hemispheres) predict attention to fearful faces as measured during eye tracking. Here, it is important to note that the fNIRS measurement took place right before the eye tracking measurement during the same experimental session at 7 months of age (see Fig 5 for details regarding the fNIRS measurement, S2 Fig for the hemodynamic response functions, and S2 Table for a group comparison between emotions). Our regression analysis revealed that only the left dlPFC responses when viewing fearful faces while measuring fNIRS predicted attentional bias to fearful faces, measured through eye tracking, *ß* = 0.261, *t* = 2.098, *p* = 0.04; *R*^*2*^ = 0.068. Specifically, greater left dlPFC responses during the fNIRS measurement were associated with an increased attentional bias to fearful faces during the eye tracking task. No such effects were found when using attentional bias to fearful eyes or duration of first look to fearful faces as dependent variables in the regression.

We then tested whether brain responses to fearful faces in left dlPFC at 7 months also predicted helping behavior at 14 months, but this association failed to reach significance, *ß* = −0.222, *t* = −1.685, *p* = 0.098. When controlling for maternal engagement as assessed during free play (see Materials and methods), which was found to be significantly and negatively associated with left dlPFC responses to fearful faces (see results concerning maternal factors presented below), brain responses to fearful faces in left dlPFC at 7 months were significantly (negatively) associated with helping behavior at 14 months, *r* (57) = −0.272, *p* = 0.037 (see Table 1; also note that here we report the statistics from a partial correlation analysis controlling for maternal engagement and that the degrees of freedom value is smaller because we did not have maternal engagement data from all 64 infants who contributed to the fNIRS and helping data).

To be consistent with our eye tracking analysis reported above, we carried out an additional analysis in which we relied on helping behavior coded as a binary measure (helping versus no helping) (see Table 2). Using logistical regressions, this analysis revealed that right dlPFC responses to fearful faces significantly predicted the binary helping measure, *b* = −0.142, χ^*2*^ *(1) =* 5.235, *p* = 0.022; *R*^*2*^ = 0.111, whereas the left dlPFC response to fearful faces was not significantly associated with the binary helping measure, *p* = 0.486. The right dlPFC response to fear was not significantly associated with any of the eye tracking responses to fearful faces/eyes.

In order to explore whether it is the differential brain response to fearful expressions when compared to other negative facial expressions that accounts for differences in helping behavior, we computed a differential brain response variable by subtracting the brain response to angry faces (as a control) from the response to fearful faces in the dlPFC. This analysis based on the discriminatory brain response that distinguishes fear from anger revealed that the right dlPFC at 7 months significantly predicted helping behavior at 14 months (*ß* = −0.335, *t* = −2.756, *p* = 0.008), indexing that a greater right dlPFC response to fearful faces compared to angry faces is associated with reduced helping behavior (note that no such association was seen when assessing left dlPFC response, *p* = 0.292) (Table 1). This prompted us to assess whether the discriminatory response to fearful faces in the right dlPFC is also associated with variability in the reported eye tracking measures. This exploratory analysis showed that right dlPFC response (fearful minus angry) was significantly associated with differences in the duration of the first look to fearful eyes (*ß* = −0.257, *t* = −2.08, *p* = 0.042), indexing that greater discriminatory right dlPFC responses to fearful faces were associated with reduced duration in first looks to fearful eyes.

Note that for the fNIRS findings reported above, only the result concerning the association between the discriminatory brain response that distinguishes fear from anger revealed in right dlPFC and altruistic behavior would survive the adjustment of the *p-*value threshold to *p* < 0.01.

### Exploratory analysis of maternal factors, fearful temperament, and cortisol

Our analysis further revealed that left dlPFC responses were predicted by maternal engagement, measured when infants were 5 months of age (see S3 Fig). Specifically, maternal engagement was negatively associated with left dlPFC responses to fear, with higher maternal engagement linked to reduced left dlPFC responses to fear (*ß* = −0.340, *t* = −2.757, *p* = 0.008), whereas there was no such association with the dlPFC response to fearful faces in the right hemisphere, *p* = 0.862. The association between maternal engagement and left dlPFC responses to fear holds when controlling for maternal age and education by including them as predictors in the regression, *ß* = −0.339, *t* = −2.661, *p* = 0.010 (maternal age and education: *ß* = 0.029, *t* = 0.219, *p* = 0.827 and *ß* = −0.010, *t* = −0.078, *p* = 0.938, respectively). Moreover, maternal engagement itself was not directly associated with helping behavior, *p* = 0.648. In order to test for the possibility that left dlPFC response to fear at 7 months moderates the association between maternal engagement at 5 months and altruistic behavior at 14 months, we employed Hayes’s PROCESS model. This analysis did not provide evidence for a moderation (interaction) effect between left dlPFC and maternal engagement (*p* = 0.763) but showed that left dlPFC responses were positively associated with helping behavior when controlling for maternal engagement (see fNIRS results presented above). Infant fearful temperament, measured through parental report at 7 months of age, when the fNIRS measurement took place, did not have an effect on dlPFC responses to fearful faces in infants. Furthermore, we did not observe any effects of cortisol on infant and maternal measures.

## Discussion

The current study shows that responses to fearful faces at 7 months, but not happy or angry faces, predict altruistic behavior at 14 months of age. This confirms our hypothesis based on prior work [7, 8, 11] and suggests that the tendency to engage altruistically is linked to responding to others in distress. This finding supports accounts that assign a critical role to other-oriented and care-based responding in human cooperation [4, 6]. Our results further show that variability in the tendency to sensitively respond to others in fear (a) emerges early in human ontogeny and (b) manifests itself in differences in the recruitment of a prefrontal brain region linked to cognitive and attentional control of emotions. This is in line with the notion that a caring continuum exists, along which individuals differ in their capacity to display sensitive responses to others’ distress that motivate prosocial action [6–8]. In summary, the current findings provide novel insights into the developmental and brain precursors of human altruism by uncovering its early ontogenetic predictors.

The current study was designed to examine responsiveness to fearful faces during a period in ontogeny when sensitivity to fear in others first comes online, offering a unique opportunity to shed light on precursors of altruistic behavior in human development. The current developmental data are important also because responsiveness to fear in others is thought to be a marker of (or precursor to) empathic concern, which has been shown to be systematically linked to altruistic behavior in older infants and adults [6, 31, 32]. In support of this idea, our study found that, at age 7 months, variability in neural responses and attentional responses to fearful faces as a display of distress predicts altruistic helping behavior at age 14 months. Our data show that responsiveness to fearful faces, but not happy or angry faces, predicts prosocial behavior. This is in line with existing work in adults [6] and demonstrates that fear processing is linked to prosocial action from as early in ontogeny as it can be experimentally investigated. Nonetheless, it is critical to mention that other negative facial expressions might also engender empathic concern and be linked to prosocial tendencies in the observer [6]. Another facial expression that has been extensively used in research on empathic responding in adults is pain [33]. Yet infants, while able to discriminate painful faces from similar-looking negative facial expressions, such as anger, at 8 months of age, do not show the brain signatures indicative of empathic responding to pain seen in adults [34]. The current study shows that responding to fear might represent a particularly early-developing other-oriented empathic process linked to prosocial tendencies. Nevertheless, in future research, it will be essential to extend the current approach by including other expressions that control for novelty and emotional expressions, such as sadness, that have been shown to elicit prosocial behavior in children [35]. This will be important in order to determine whether the effects observed in the current study generalize to novel or other concern-inducing expressions or are indeed specific to fear.

With respect to our eye tracking results, we observed that 7-month-old infants, who initially showed heightened attention (prolonged first look) but then more effectively disengaged from fearful faces (reduced looking bias) when shown over a prolonged period of time (15 seconds), display higher levels of altruistic behavior as 14-month-old toddlers. Our eye tracking data further showed that, more specifically, effective disengagement from fearful eyes (reduced looking bias) predicted greater levels of altruistic behavior. This is noteworthy insofar as wide-open eyes and large eye whites have been shown to play a pivotal role in fear detection in infants and adults [36, 37]. The observed pattern of infants’ initial heightened attentional engagement to fearful faces followed by longer-term disengagement from the fearful faces and especially eyes may represent a mechanism that helps facilitate prosocial action in the individual. In line with this pattern observed among infants in the current study, previous research shows that altruistic behavior likely arises from a combination of initial shared distress (arousal) with the other, followed by a down-regulation of one’s own distress to enable prosocial behavior [38]. Indeed, unsuccessful disengagement from others’ distress results in what has been termed as personal distress—a self-oriented response characterized by negative affect—which is linked to the tendency to escape the situation rather than help [39, 40]. The current infant eye tracking data are therefore broadly consistent with previous research and present an intriguing view into the attentional and possibly regulatory (cognitive control) mechanisms that shape individual variability in prosocial functioning from early in ontogeny.

Moreover, we were able to corroborate the obtained eye tracking findings when helping behavior is coded as a binary measure (helping versus no helping) rather than as a continuous measure (helping score 0 through 6), as done for the findings presented and discussed above. Our analysis shows that duration of the first look both to fearful faces and to fearful eyes significantly predicted helping behavior. These results from using a binary helping measure principally confirm the results from our analysis using a continuous measure of helping and point to initial heightened attention (first look) to fearful faces and eyes as the best predictor of whether an infant does or does not help at age 14 months. In combination with the findings presented above, this also suggests that while initial heightened attention to fear (distress) in others is linked to whether an infant helps or not, repeatedly offering help to a person in need may depend more on an infant’s regulatory abilities, reflected in effectively disengaging from others in distress once detected.

With respect to our analysis of infants’ brain responses to emotional facial expressions using fNIRS, we observed that infants’ left dlPFC responses predicted their attention to fearful faces (looking bias), measured using eye tracking, which directly followed the fNIRS recording. Specifically, our data show that greater involvement of left dlPFC was linked to a heightened attentional looking bias (increased looking time) to fearful faces. Moreover, our fNIRS analysis indexed that left dlPFC at 7 months of age is not only associated with attentional bias to fearful faces but also predicts altruistic helping behavior at 14 months, with reduced recruitment of left dlPFC being predictive of greater levels of altruistic behavior (note that the association between infants’ left dlPFC responses and their helping behavior as toddlers was observed when controlling for maternal engagement, which was also found to be associated with left dlPFC responses to fear; for discussion, see below). In order to better interpret this finding, it is important to remember that, in adults, greater amygdala responses to fearful faces were linked with greater levels of altruistic behavior [8] and that previous work shows that greater dlPFC involvement is linked to reduced amygdala activity [22, 41]. The current results raise the possibility that a similar inverse functional relation between dlPFC and amygdala may exist in infants. However, it should be emphasized that fNIRS did not allow us to measure amygdala responses, and prior work with children and adolescents suggests that inverse coupling between the prefrontal cortex and amygdala does not mature until much later in development than in the current study [42]. In this context, it is interesting to note that left dlPFC responses predicted reduced attention to fearful faces when measured over the course of the entire duration of the face presentation trial (15 seconds), whereas the initial heightened attention to fearful eyes, measured as the duration of the first look, was not explained by left dlPFC. One possible interpretation of the current fNIRS data is that less engagement of dlPFC may represent more effective cognitive or attentional control (disengagement) when responding to fear in others, which might be required in order to initiate prosocial actions when seeing others in need or distress. This interpretation is in agreement with work suggesting that (emotion) regulation and control play a key role in empathic concern and prosocial responding [27, 39, 43].

Moreover, this interpretation is particularly relevant considering that fearful faces were presented repeatedly (5 times) within each fNIRS trial (see Materials and methods), suggesting that reduced left dlPFC responses may reflect more effective neural habituation (repetition suppression) within this brain region to fearful faces. This may also help explain why infants with reduced left dlPFC responses, indexing greater neural habituation, then showed a reduced looking bias to fearful faces in the eye tracking task, indicating greater attentional habituation (note that the eye tracking did not employ the same procedure of showing repeated presentations of a single facial emotional expression; see Materials and methods). Clearly, this is only a tentative proposal of how to interpret the fNIRS findings, and future research should more directly examine infants’ brain responses to repeatedly presented images of facial emotional expressions.

Additional exploratory analyses showed that infants’ brain responses in the right dlPFC, but not in the left dlPFC, predicted helping behavior when coded as a binary outcome (help versus no help), whereby reduced right dlPFC responses to fearful faces were associated with a greater likelihood of helping among infants. This association between right dlPFC and helping behavior was also seen when a difference measure (fearful minus angry) was used as a predictor of continuous helping behavior. Together, this suggests that, rather than being lateralized, it is activity within regions in the left and right dlPFC that accounts for variability in fear processing and its link to prosocial action tendencies. This is largely in line with a body of research with adults indicating that it is an interplay of left and right dlPFC regions that is linked to both exerting cognitive control and driving motivational tendencies when processing emotional information [41, 44, 45].

It is striking to observe dlPFC involvement in fear processing and prosocial responding at such an early stage in ontogeny. This is especially true considering research showing the protracted structural and functional development of dlPFC, which extends well into adolescence and early adulthood [43, 46]. More specifically, there is evidence from functional MRI (fMRI) research showing that left dlPFC plays an important role in self-control during cooperative decision-making and develops only gradually in school-aged children [43]. The current data suggest that this brain region is also implicated in perhaps more basic processes linked to early-developing forms of cooperative behavior. This is in line with the emerging view that, other than previously assumed, the prefrontal cortex is functionally involved in shaping emotion, cognition, and behavior from infancy [47, 48].

Our results further showed that variability in infants’ left dlPFC responses to fearful faces at 7 months is predicted by maternal factors at 5 months of age. We observed that lower maternal engagement with her infant during free play at 5 months predicts greater left dlPFC responses to fearful faces at 7 months. This provides preliminary evidence for an effect of maternal engagement on dlPFC function during emotion processing in infants. It remains an open question how exactly these maternal variables impact infant emotional brain function. One possibility is that reduced maternal sensitivity [49], which has been shown to be linked to less effective responding to fear and distress of the infant, does not provide infants with sufficient experience of regulating—or rather, coregulating—their own distress and fear. This might then in turn impact how infants perceive and respond to others when displaying distress and fear [49]. Most importantly, with respect to our analysis of predictors of altruistic behavior, we could show that left dlPFC responses at 7 months predict helping behavior at 14 months when controlling for maternal engagement measured at 5 months. This suggests that while maternal engagement is associated with variability in left dlPFC function during fearful face processing, it does not account for its association with helping behavior. This finding is important in light of accumulating evidence showing that socialization may play a role in the display of helping behavior in toddlers [29, 30], as it indicates that, at least as far as maternal engagement and sensitivity during early months are concerned, the link between fear processing and prosocial behavior exists independent of this maternal factor.

In summary, the current study provides novel insights into the nature of human altruism by uncovering predictors of prosocial behavior in early human development. The present findings support the notion that responsiveness to fear in others can be seen as a key marker and ontogenetic predictor of prosocial action among humans. Our results indicate that variability in attention and dorsolateral prefrontal brain responses to fearful faces and eyes exists at 7 months of age, which is linked to the level of altruistic behavior displayed at 14 months of age. Taken together, this paints a much-needed integrative picture of the early ontogeny of prosocial behavior, thereby advancing a more mechanistic understanding of the emergence of human altruism.

## Materials and methods

### Ethics statement

The study was approved by the Ethics Committee at the Medical Faculty, Leipzig University (236-10-23082010), and was conducted in accordance with the Declaration of Helsinki.

### Participants

Sixty-four infants (30 females) participated in this study at 3 time points: 5 months (*M*_*age*_ = 147.39 days, SD = 15.16), 7 months (*M*_*age*_ = 214.81 days, SD = 6.79), and 14 months (*M*_*age*_ = 432.30 days, SD = 8.70). Mothers of these infants also participated at the 5-month visit (*M*_*age*_ = 31.56 years, SD = 4.07). All infants were of European descent, were born at standard gestational age (over 38 weeks), and had a normal birth weight (>2,500 grams). Parents provided written informed consent prior to participation and were compensated with travel money, a photograph of the infant, and a toy at each visit.

### Cortisol measurement at 5 months

Mother–infant dyads were instructed to abstain from eating or drinking anything apart from water 30 minutes prior to their appointment. Sessions were most frequently held in the late morning to account for diurnal cortisol changes (mean arrival time = 11:00, SD = 1:52). Saliva samples were collected from infants and their mothers about 5 minutes post arrival using commercially available devices (the Salimetrics Infant’s Swab [Salimetrics, Suffolk, UK] and the Salivette [Sarstedt, Nümbrecht, Germany], respectively). Samples were stored in a −80 °C freezer after each experimental session and were later transported at room temperature for analysis. Cortisol levels were analyzed at the Technical University of Dresden, Germany, using luminescence immunoassay kits purchased from IBL International (Hamburg, Germany). The functional sensitivity of the assay was 0.011 (micro)g/dL. Inter- and intra-assay coefficients of variation were <8%. Raw cortisol levels were log-transformed to correct for positive skew.

### Free play social interaction at 5 months

The free play interaction occurred after saliva collection. In order to capture a natural play session, each mother was instructed to interact with her infant as she did at home. No further instructions were given. Infants were put on their backs on a soft blanket. Two cameras were set up to record the interaction simultaneously: one camera captured the face and body of the mother, and the other camera captured the face and body of the infant. Mothers were aware of the cameras and were instructed to remain within view. The same selection of 5 objects (4 toys and 1 play book) was provided to each mother to assist play. Mothers could freely choose and change the object of interest during the interaction (or abandon the objects altogether) to suit the infants’ needs and preferences. Once assembled, the experimenter left the room, and the mother–infant pair was left alone to interact for 5 minutes.

#### Free play analysis

Video recordings of mothers and infants were aligned using the open-source software Kdenlive. This generated a single time-locked video of the entire interaction. The first seconds of each video were cropped to create an exact 4-minute time window from which to code. One trained (primary) coder assessed each interaction based on a coding scheme previously developed in-house to assess maternal and infant engagement [50]. The coding was designed to capture behaviors from mothers (verbal engagement, physical proximity to infant, visual attention/engagement toward infant, and positive mood) and infants (visual attention/engagement toward mother and positive mood) to be answered on a scale from 1 (very low) to 5 (very high). Additional factors were coded within INTERACT Software (Mangold International), including duration of physical touch and infant smiles and laughter. To test reliability of the primary coder, an additional trained coder assessed a random subsample (25%) of the videos. Interrater reliability was very high (Chronbach’s α = 0.855). Z-scores were generated for each variable and were averaged to create composite scores of maternal engagement (verbal engagement, physical proximity to infant, visual attention/engagement toward infant, positive mood, and touch) and infant engagement (attention/engagement toward mother, positive mood, smiles, and laughter).

### fNIRS at 7 months

The 7-month visit consisted of an fNIRS experiment followed by an eye tracking experiment. Additionally, mothers filled out the Infant Behavior Questionnaire in its revised form (IBQ-R) [51]. This questionnaire provides information regarding various temperament traits of infants. For our analysis, we particularly focused on the fearfulness of the infant. This was done because prior work indicates that infant fearfulness correlates with frontal brain responses to fearful (body) expressions [52]. The methods of the fNIRS and eye tracking experiments conducted during this visit are detailed below.

#### Stimuli

Color photographs of 5 Caucasian females expressing happiness, anger, fear, and neutrality were chosen from a validated and published stimulus set (FACES Collection) [53]. The 5 actresses chosen had expressions with average recognition accuracies at or above 93.25%. Using Adobe Photoshop CS5, faces were placed within (below) a predetermined oval in the center of a light gray background. Photographs were moved and resized to align with fixed markers for the position of the 2 eyes, the mouth, and the nose. This editing technique removed any potentially distracting outer features of the faces, such as the ears and hair, and also ensured that all inner facial features were presented in approximately the same location on the screen. Baseline images consisted of color photographs of 5 inanimate objects (vegetables) presented in the center of a light gray background (same background as chosen for the face stimuli). The inanimate object images have been used as baseline stimuli in previously published infant fNIRS studies concerned with infant face processing [19, 54, 55]. The visual angles of the facial stimuli and inanimate baseline stimuli were around 15.7° × 21.7° and 16.8° × 16.8°, respectively. A 3-second video clip of a shaking rattle accompanied by sound (from Tobii software, Sweden) was used as an attention getter to orient infants to the center of the screen. The rattle had an approximate visual angle of 4.6°, and the sound comprised 3 alternating frequency tones (from 109 Hz to 262 Hz).

#### Procedure

Infants were seated on their parent’s lap in a quiet, dimly lit room, facing a screen (52 cm × 32 cm) at a distance of approximately 60 cm. The monitor was adjusted such that the center of the screen was approximately at eye level for the infants, which ensured that the visual stimuli could be easily detected while making it difficult for the parents to see the screen. Moreover, parents were instructed to avoid watching the screen and make sure that their infants did not touch the fNIRS cap throughout the experiment. A room divider separated the experimental area from the control desk. As in prior published studies [50, 56], a small plastic ring was provided for each infant to hold during the experiment, since this has proven to reduce arm and body movements during the experiment. A camera at the bottom of the screen tracked infant behavior online and allowed for offline coding of attention during each trial of the experiment. Stimuli were presented using Presentation software (Neurobehavioral Systems, United States).

The experimental paradigm consisted of blocks of 3 randomized trials each of happiness, anger, and fear. Each block began with an attention getter to keep infants alert and to orient them to the center of the screen (see Stimuli above). Each trial consisted of a baseline inanimate object stimulus presented for at least 6 seconds, followed by an emotional face stimulus for 6 seconds. A brief 150-ms bell sound (about 600 Hz) preceded each baseline and emotional face presentation to orient infants’ attention to the screen. Facial identities were pseudorandomized such that each infant viewed every possible actress–emotion combination, no actress was shown expressing the same emotion consecutively, and no emotional expression was repeated more than once in a row. Similar pseudorandomization parameters were used for the inanimate baseline stimuli such that no image served as the baseline twice in a row, and every possible baseline image–emotion combination was presented.

Both the baseline and face stimuli were presented in a way to create a dynamically changing visual stimulation. The baseline was composed of at least 6 seconds of the same photograph changing from its original size (500 ms) to a slightly larger size (approximately 1° increase in visual angle) (700 ms) at least 5 times. The face presentation consisted of exactly 6 seconds of the same actress changing from a neutral expression (500 ms) to the target emotion (700 ms) 5 times (see Fig 5). This method of pseudodynamic presentation of facial expressions was adapted from previous infant fNIRS paradigms from Nakato and colleagues [57] and ensured that infants maintained attention during the relatively long trials that fNIRS measurement requires. Infants viewed an average of 30.75 total trials (range = 18–43; SD = 5.847).

Infant fNIRS data were recorded using an NIRx NIRScout system (Germany) and NIRStar acquisition software. fNIRS data were recorded from 32 optodes (16 sources, 16 detectors) placed at an approximately 2.5-cm distance within an elastic cap (EasyCap, Germany) typically used for EEG measurements with infants in other studies but customized for the purpose of infant fNIRS studies focused on localizing brain responses in frontal and temporal cortices (see [58] for a prior infant fNIRS study using this customized cap and channel layout). Identical to how the cap is applied in EEG research, the cap containing the optodes was positioned on the infant head according to anatomical landmarks (using nasion, inion, and left and right mastoids as reference points). This arrangement created 49 channels (source–detector pairs) placed over frontal and temporal cortices in both hemispheres (see Fig 5 for channel layout and cortical projections determined by the nirsLAB analysis package [NIRx] and shown with reference to the 10–20 electrode positioning system). Data were recorded at a sampling rate of 6.25 Hz. Near-infrared light was emitted at 2 wavelengths (760 nm and 850 nm), with a power of 5 nm/wavelength. The system automatically adjusted light intensity in order to provide optimal gain.

#### Data analysis

Videos from each individual session were initially coded for looking duration to each trial. Trials were only included in the fNIRS analyses if infants had attended to the screen for at least 4 of the 6 seconds in which both baseline and face stimuli were presented. Additionally, the fNIRS data were visually inspected for motion artifacts. Trials with motion artifacts were completely removed from further analyses. The remaining cleaned data were analyzed using the Matlab-based software Nilab2 (NIRx, Germany). Data were filtered with a 0.2-Hz low-pass filter in order to remove fluctuations that were too fast (i.e., fluctuations due to heart rate) and a high-pass filter of 12 seconds in order to remove changes too slow to be related to experimental stimuli (i.e., fluctuations due to drift). Using 6-second time windows after face onset (equaling the stimulus presentation duration of each emotional face), measurements were converted into oxygenated hemoglobin (oxy-Hb) and deoxygenated hemoglobin (deoxy-Hb) using the Beer-Lambert law. The average concentration changes of oxy-Hb and deoxy-Hb in response to each emotional expression were extracted for each channel for each individual infant. All concentration change values were scaled (×1,000) in an effort to avoid working with small decimals. All infants provided at least 3 artifact-free trials per emotion (happy trials: *M* = 7.14, SD = 2.34; angry trials: *M* = 7.09, SD = 2.16; fearful trials: *M* = 6.52, SD = 2.26). Our analysis specifically focused on responses within brain regions in the superior temporal, inferior frontal, and dorsolateral frontal cortices (see Fig 5). These regions of interest were created a priori on the basis of the cortical projections of our fNIRS channels onto MNI space computed in the nirsLAB analysis package (NIRx) and with reference to 10–20 EEG electrode placement and its known cortical projections in infants [59].

### Eye tracking at 7 months

#### Stimuli

Photographs of 3 out of the 5 female actresses used in the fNIRS experiment displaying happy, fearful, angry, and neutral facial expressions were chosen for creating the stimuli for the eye tracking experiments. Stimuli were created in Adobe Photoshop Version CS5 such that each emotional face was presented side by side with the neutral face of the same actress. Each face had a visual angle of about 15.7° × 21.7° (see [16] for details).

#### Procedure

Eye tracking occurred immediately after the fNIRS paradigm. Infants remained seated on their parents’ lap while the fNIRS cap was removed. Stimuli were presented through Tobii Studio (Version 3.2) on the same monitor on which the fNIRS stimuli were viewed. A Tobii X120 eye tracker attached to the bottom of the computer monitor recorded infant looking behavior. Prior to stimulus presentation, a 5-point calibration procedure was administered. The calibration procedure was repeated for infants that did not successfully fixate.

Each infant viewed a total of 9 trials, with each of the 3 emotions presented 3 times, once by each of the 3 actresses. Stimulus presentation was pseudorandomized such that no emotion and no actress were repeated in successive trials. Moreover, the side (left or right) on which the emotional face was presented was counterbalanced for each infant. Infant behavior was recorded online such that the experimenter could assess attention and had full control over the presentation of each trial. Each trial began with a 3-second attention getter in the center of the screen. Experimental trials were presented for 15 seconds (see Fig 2). During that period, infants were able to freely explore the faces and look towards and away from the screen.

#### Data analysis

Areas of interest (AOIs) for faces and eyes were created within Tobii Studio (see Fig 2 for an illustration of the face and eye AOI definition). For these 2 AOIs, similar to previous research, we focused our analysis on 2 eye tracking measures: (a) duration of the first look to emotion and (b) overall looking bias to emotion [11, 56]. While the duration of the first look is directly computed by the Tobii eye tracking software, for the calculation of the overall looking bias to emotion, we undertook the following steps. The total looking times to face and eyes were extracted for each emotional and neutral stimulus per infant, per trial. As reported before [16], looking bias scores were calculated by taking the proportion of looking time to each emotion (anger [A], fear [F], happiness [H]) as compared to its corresponding neutral face (neutral [A], neutral [F], neutral [H]). The attentional bias for any given emotion (X) was computed as follows:

$$attentional\;bias\;(X)\;=\;\frac{looking\;time\;(emotion\;X)}{looking\;time\;(emotion\;X)+looking\;time\;(neutral\;X)}$$

We were therefore able to compare the percentages of looking time towards anger, fear, and happiness while taking into account the time the infant spent looking at neutral stimuli. The visual inspection of heat maps throughout the session revealed that infants fixated on the moving rattle during all 9 attention getters, indicating firstly that there was no drift across the experimental session and secondly that infants maintained central looking before the presentation of the facial stimuli.

Note that we removed outliers from the variables reported as significant predictors at 5 and 7 months using the interquartile range rule (IQR) [60].

### Prosocial behavior at 14 months

Instrumental helping behavior was assessed using paradigms adapted from a previously published study [2]. The experiment consisted of 2 situations in which an experimenter required assistance obtaining an object that was out of reach. Each situation was presented 3 times, resulting in a total of 6 opportunities for the infant to help. In the first situation (PEN), the experimenter sat at a table and drew a picture. The infant was placed on the ground in front of the table so that she could view the experimenter’s actions. The experimenter then dropped her pen, making it appear accidental. For the first 10 seconds, the experimenter looked at and reached for the pen, but never touched it. In the next 10 seconds, the experimenter continued to reach for the pen but switched eye contact between the infant and the pen. In the last 10 seconds, the experimenter reached, switched eye contact, and said “Oh, my pen!”. This procedure was repeated twice, resulting in 3 trials for this situation. The entire paradigm was video recorded and coded for infant helping frequency (the number of trials the infant picked up the pen and handed it to the experimenter) and latency (the duration between the pen hitting the ground and the moment the pen was handed back to the experimenter). The second situation (BALL) involved the experimenter and infant (on the parent’s lap) seated at a table, facing each other. Three paper balls were in front of the experimenter (and out of the infant’s reach), and 3 balls were located in front of the infant. Using tongs, the experimenter picked up the 3 balls in front of her and placed them in a box. The experimenter then reached for one of the balls on the infant’s side but could not reach it. The experimenter looked at and tried reaching for the ball for 10 seconds. In the next 10 seconds, the experimenter continued to reach for the ball, switching eye contact between the ball and the child. In the next 10 seconds, the experimenter continued to reach, switch eye contact, and say “Oh, my ball!”. This procedure was repeated twice, resulting in 3 trials for this situation. Again, trials were coded for helping frequency (number of trials helped out of 3 trials) and for the latency to help. Sessions were video recorded with 3 cameras, and all of the sessions were coded by a primary coder who was not involved in the experiment and was blind to the hypotheses. A random sample of 25% of the sessions were coded by a secondary coder to establish intercoder reliability. Coding for the target behavior (i.e., did the infant hand over the pen) achieved perfect reliability, *κ* = 1. Concerning the helping tasks (situations) used in the current study, it is critical to mention that in the study our helping tasks were based on, appropriate control conditions were used, ascertaining that infants at the age of 14 months detect situations when another person needs help [2]. We decided to adapt selected experimental conditions from this previous study because we intended to investigate variability in helping behavior and whether it can be predicted by emotion processing measured earlier in infancy. Our initial analysis relied on 2 variables computed on the basis of the infants’ performance in the 2 helping tasks: (a) frequency of helping behavior displayed across the 2 tasks, which could range from 0 to 6, and (b) latency of helping behavior averaged across trials. However, since 24 out of the 64 infants included in the study did not provide any help, we focused our analysis reported in the Results section on the frequency rather than the latency measure in order to avoid dropping a large proportion of our sample from the analysis. Infant helping frequency ranged from 0 to 6 trials (*M* = 1.83, SD = 1.95).

## Supporting information

S1 Fig This figure shows the distributions of the eye tracking measures. Plotted are the kernel density distributions of our 2 eye tracking measures for each of the 3 emotional face conditions. Note that density plots are variations of histograms that use kernel smoothing to visualize the distribution of data over a continuous time period or interval and have the advantage of better capturing the shape of distributions because they do not depend on bin widths. Please note that infants viewed photographic images of real faces (see Materials and methods) and that the facial images shown here were computer generated with the FaceGen software (https://facegen.com) for illustrative purposes. Underlying data are available through the Open Science Framework, https://osf.io/znjr7/.
(TIF)

S2 Fig This supplementary figure shows the hemodynamic response (oxy-Hb) to the emotional faces (happy, angry, and fearful) for the following regions of interest: dlPFC = blue; IFC = violet; STC = green; TPJ = red. dlPFC, dorsolateral prefrontal cortex; IFC, inferior frontal cortex; oxy-Hb, oxygenated hemoglobin; STC, superior temporal cortex; TPJ, temporo-parietal junction.
(TIF)

S3 Fig This supplementary figure shows the association between maternal engagement coded from a free play interaction with the infant at 5 months of age and infant brain responses to fearful faces at 7 months in the dlPFC. The maternal engagement score was composed of coded maternal behaviors concerning verbal engagement, attention, positive mood, proximity, and touch. Underlying data are available through the Open Science Framework (https://osf.io/znjr7/). dlPFC, dorsolateral prefrontal cortex.
(TIF)

S1 Table This shows the means and standard errors of the eye tracking measures for the 3 emotional face conditions. The statistics column displays results from a repeated-measures ANOVA to investigate the main effect of emotion. Note that when sphericity could not be assumed, Greenhouse-Geisser values are reported. Underlying data are available through the Open Science Framework, https://osf.io/znjr7/.
(DOCX)

S2 Table This shows the means and standard errors for the 3 emotional face conditions within the brain regions of interest. The statistics column displays results from a repeated-measures ANOVA to investigate the main effect of emotion. Note that when sphericity could not be assumed, Greenhouse-Geisser values are reported. Underlying data are available through the Open Science Framework, https://osf.io/znjr7/.
(DOCX)

## Abbreviations

AOI: area of interest
deoxy-Hb: deoxygenated hemoglobin
dlPFC: dorsolateral prefrontal cortex
EEG: electro-encephalography
fMRI: functional MRI
fNIRS: functional near-infrared spectroscopy
IBQ-R: Infant Behavior Questionnaire in its revised form
IQR: interquartile range rule
oxy-Hb: oxygenated hemoglobin
TPJ: temporo-parietal junction
