# Supplementary figures and images for: The neurodevelopmental precursors of altruistic behavior in infancy
Source: PLoS Biol. 2018 Sep 25;16(9):e2005281. doi: 10.1371/journal.pbio.2005281 (PMC6155440; doi:10.1371/journal.pbio.2005281)

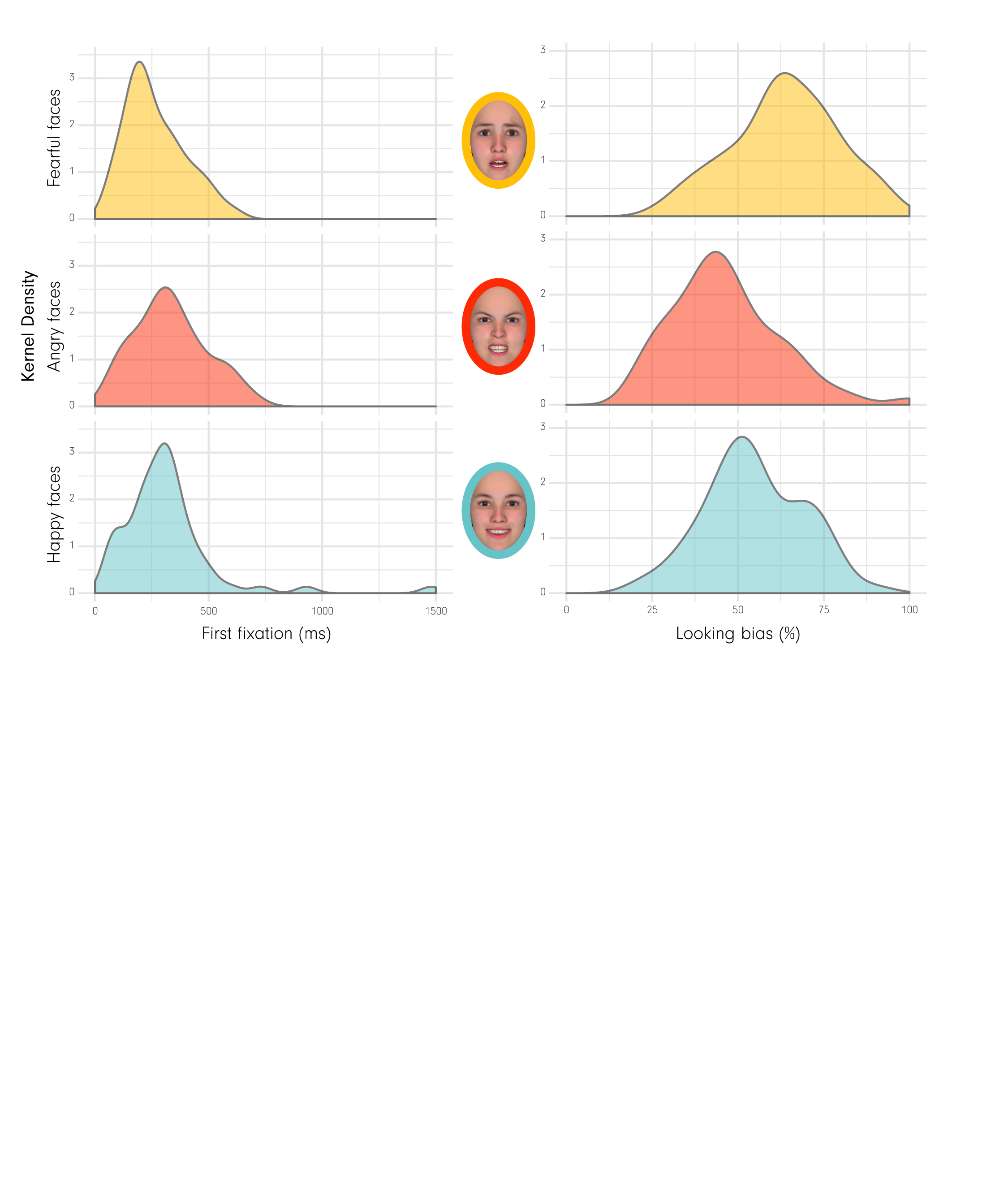

Supplement: S1 Fig — Plotted are the kernel density distributions of our 2 eye tracking measures for each of the 3 emotional face conditions. Note that density plots are variations of histograms that use kernel smoothing to visualize the distribution of data over a continuous time period or interval and have the advantage of better capturing the shape of distributions because they do not depend on bin widths. Please note that infants viewed photographic images of real faces (see Materials and methods) and that the facial images shown here were computer generated with the FaceGen software (https://facegen.com) for illustrative purposes. Underlying data are available through the Open Science Framework, https://osf.io/znjr7/. (TIF) [file pbio.2005281.s001.tif]

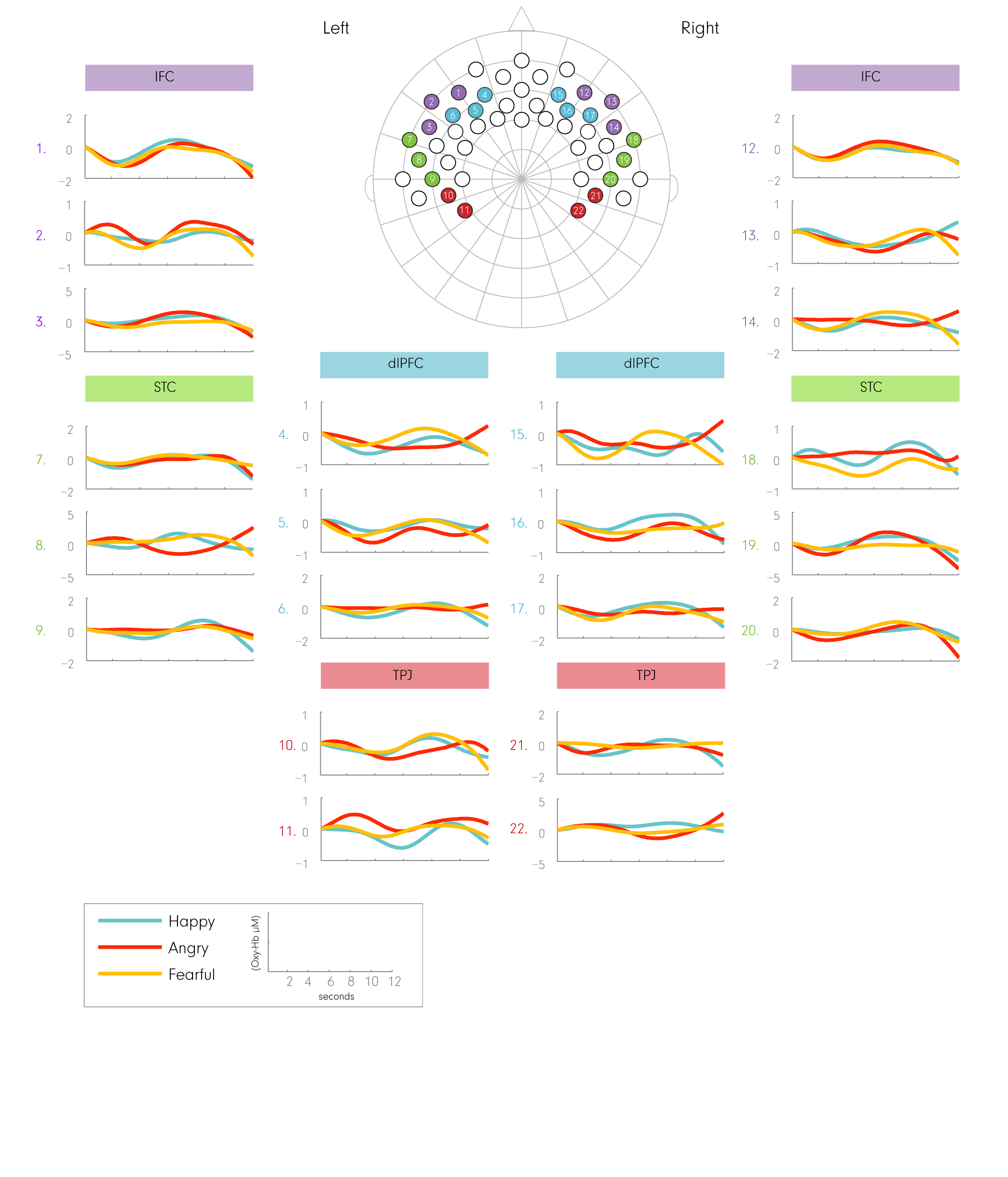

Supplement: S2 Fig — dlPFC, dorsolateral prefrontal cortex; IFC, inferior frontal cortex; oxy-Hb, oxygenated hemoglobin; STC, superior temporal cortex; TPJ, temporo-parietal junction. (TIF) [file pbio.2005281.s002.tif]

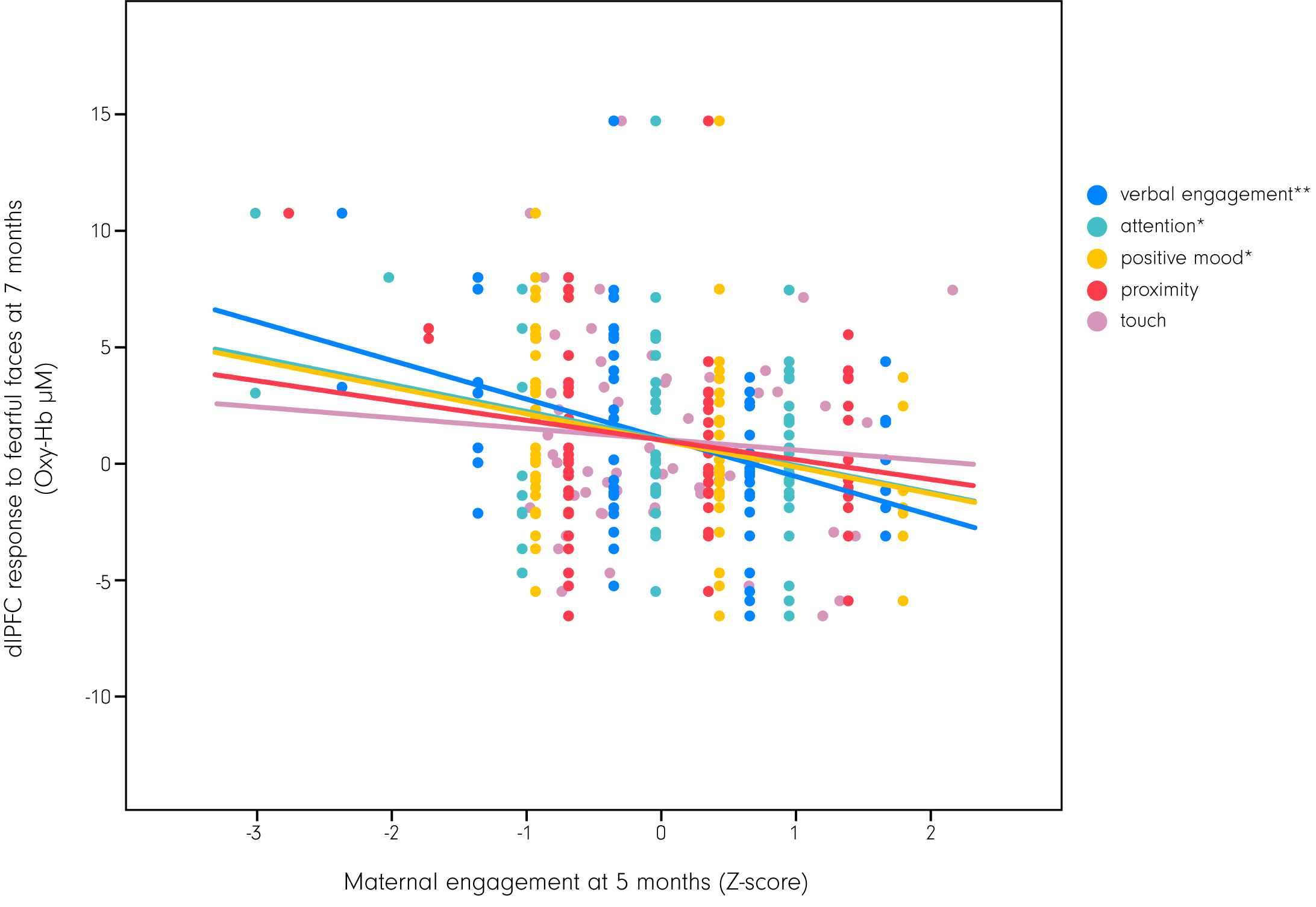

Supplement: S3 Fig — The maternal engagement score was composed of coded maternal behaviors concerning verbal engagement, attention, positive mood, proximity, and touch. Underlying data are available through the Open Science Framework (https://osf.io/znjr7/). dlPFC, dorsolateral prefrontal cortex. (TIF) [file pbio.2005281.s003.tif]
