# Supplementary material for: The neurodevelopmental precursors of altruistic behavior in infancy
Source: PLoS Biol. 2018 Sep 25;16(9):e2005281. doi: 10.1371/journal.pbio.2005281 (PMC6155440; doi:10.1371/journal.pbio.2005281)
Supplement: S1 Table — The statistics column displays results from a repeated-measures ANOVA to investigate the main effect of emotion. Note that when sphericity could not be assumed, Greenhouse-Geisser values are reported. Underlying data are available through the Open Science Framework, https://osf.io/znjr7/. (DOCX) [file pbio.2005281.s004.docx]

|  | Fear  Mean (SE) | Anger  Mean (SE) | Happiness  Mean (SE) | Statistics |
| --- | --- | --- | --- | --- |
| Looking bias (%) | 63.78 (1.95) | 46.39 (1.97) | 54.72 (1.80) | *F* (2, 126) = 19.60, *p* = 0.00000004 |
| First fixation (ms) | 258.55 (16.40) | 332.66 (19.67) | 313.60 (26.73) | *F* (1.75, 106.91) = 3.62, *p* = 0.036 |
