# Supplementary material for: The neurodevelopmental precursors of altruistic behavior in infancy
Source: PLoS Biol. 2018 Sep 25;16(9):e2005281. doi: 10.1371/journal.pbio.2005281 (PMC6155440; doi:10.1371/journal.pbio.2005281)
Supplement: S2 Table — The statistics column displays results from a repeated-measures ANOVA to investigate the main effect of emotion. Note that when sphericity could not be assumed, Greenhouse-Geisser values are reported. Underlying data are available through the Open Science Framework, https://osf.io/znjr7/. (DOCX) [file pbio.2005281.s005.docx]

|  | **Fear**  Mean (SE) | **Anger**  Mean (SE) | **Happiness**  Mean (SE) | **Statistics** |
| --- | --- | --- | --- | --- |
| **Left dlPFC** | 0.93 (0.51) | -0.33 (0.67) | 1.18 (0.54) | *F* (1.72, 106.81) = 1.81, *p* = 0.17 |
| **Right dlPFC** | 0.82 (0.67) | -0.36 (0.72) | 1.31 (1.50) | *F* (1.43, 90.14) = 0.63, *p* = 0.48 |
| **Left IFC** | 2.65 (1.83) | 0.67 (2.03) | 1.68 (1.47) | *F* (1.59, 100.05) =0.44, *p* = 0.60 |
| **Right IFC** | 2.12 (0.73) | 0.23 (0.76 | 0.28 (0.78) | *F* (2, 126) = 2.19, *p* = 0.12 |
| **Left STC** | 2.06 (1.68) | -0.52 (0.89) | 1.89 (1.17) | *F* (1.60, 100.39) = 1.53, *p* = 0.22 |
| **Right STC** | 1.93 (0.81) | 3.57 (1.65) | 3.06 (1.42) | *F* (1.50, 92.73) = 0.57, *p* = 0.52 |
| **Left TPJ** | 0.81 (0.46) | 0.13 (0.53) | 0.29 (0.49) | *F* (2, 126) = 0.57, *p* = 0.57 |
| **Right TPJ** | -0.49 (0.67) | -1.19 (1.29) | 1.24 (0.77) | *F* (1.51, 95.24) = 1.68, *p* = 0.20 |
